# Balancing Effectiveness and Ethics: Global Systematic Review of *Sus scrofa* Population Control Methods

**DOI:** 10.3390/ani16071023

**Published:** 2026-03-27

**Authors:** Jan Cukor, Monika Pařízková, Rostislav Linda, Zdeněk Vacek, Vlastimil Skoták

**Affiliations:** 1 Forestry & Game Management Research Institute, Strnady 136, 252 02 Jíloviště, Czech Republic; cukor@fld.czu.cz (J.C.);; 2 Faculty of Forestry and Wood Sciences, Czech University of Life Sciences Prague, Kamýcká 129, 165 00 Prague, Czech Republic; 3 Faculty of Forestry and Wood Technology, Mendel University in Brno, Zemědělská 1, 613 00 Brno, Czech Republic

**Keywords:** pest management, wild boar, feral pig, population reduction, overabundance

## Abstract

The rapid global wild boar (*Sus scrofa*) population growth, coupled with increasing agricultural crop damage and disease transmission, suggests that current management and control strategies remain inadequate. Therefore, an international systematic review using the Web of Science database (WoS; Clarivate Analytics, Philadepphia, PA, USA), including a quantitative synthesis (119 studies up to 11 November 2025, containing 181 experiments) of population reduction methods was conducted, with an emphasis on evaluating their effectiveness, selectivity, and animal welfare aspects relating to wild boar and feral pigs. The results demonstrate a significant increase in research interest for population control methods in recent years. The highest average effectiveness was observed for aerial shooting (56.2% of the population per month), followed by poison baiting (27.6%) and trapping (6.0%). Aerial shooting appeared highly selective in the reviewed contexts; however, together with poison baiting, it is generally not permitted under current European conditions. Trapping (6.0%) and individual hunting (3.9%) offer moderate effectiveness but are highly context-dependent. From a welfare perspective, the analysis indicated that no significant difference in effectiveness was detected between studies that included welfare or stress assessment and those that did not, indicating that consideration of animal welfare does not reduce control efficiency. The study concluded that the analysis did not identify a single universally applicable solution that combines animal welfare considerations with high effectiveness, highlighting a significant research gap. This underscores the urgent need for an effective and publicly acceptable method of reducing wild boar populations, or for the development of strategies that appropriately integrate multiple approaches. However, the interpretation of results is limited by heterogeneity in study design and variability in reported data.

## Simple Summary

The global wild boar population is rapidly increasing, causing serious damage to crops, forests, and suburban areas. The species also spreads diseases and creates growing conflicts with people. Hunting remains the main management tool; however, in many regions it has not been sufficient on its own to control population growth. In this study, we compared various methods used worldwide to reduce wild boar numbers and evaluated their effectiveness, selectivity, and consideration for animal welfare. Aerial shooting proved to be the most effective method, but it is not permitted in Europe. Poison baiting and trapping showed lower efficiency but can be applied in more contexts. Studies that included welfare assessments did not differ in effectiveness from those that did not, indicating that consideration of animal welfare does not reduce control efficiency. No single method was found to be universally suitable; however, combining several approaches appears more promising. The findings can help wildlife managers design population control strategies that are both effective and publicly acceptable.

## 1. Introduction

The wild boar (*Sus scrofa*) is one of the most successful, globally distributed mammal species. Its success is reflected in its colonization of new areas not only in Europe, but also in North and South America, Asia, and Australia [1,2]. Simultaneously, there has been a rapid increase in population densities in traditional areas of occurrence, particularly in the temperate zones of Central Europe. This trend is driven by a combination of socio-economic, ecological, and climatic factors, further exacerbated by ongoing climate change [2,3]. The wild boar is characterized by high ecological plasticity and reproductive capacity, enabling it to expand rapidly across various bioregions despite high mortality rates and changing environmental conditions [4,5,6].

One of the key factors of population growth is the intensification of agricultural practices, especially the large-scale cultivation of maize, cereals, and rapeseed, which provide sufficient shelter and abundant food resources [7,8,9]. Food availability during the summer is supplemented by a profusion of acorns and beechnuts in the non-vegetation season in areas of temperate deciduous forests, particularly in Central Europe. The ongoing climate change has contributed to more frequent and abundant mast years for beeches and oaks, thereby improving the physical condition (e.g., body weight) of females with increasing litter size and survival rates of offspring [10,11,12]. Moreover, the survival rate of subadults and the overall physical condition of wild boar have been positively influenced by increasingly mild winters, which are typical of the last few decades.

This increase in wild boar population densities has been associated with a rise in human–wildlife conflicts. In Europe, this species causes considerable damage to agricultural land, especially to energy-rich crops [13,14,15]. In some regions of Poland, wild boar have been estimated to account for up to 95% of wildlife-related damage to field crops, resulting in compensation payments amounting to millions of euros and escalating conflicts between farmers and hunters [16]. In the United States, annual economic losses caused by wild pigs are estimated to reach billions of dollars, reflecting both agricultural damage and management costs [17]. Damage to forestry can be another serious aspect, and in some local studies up to 80% of new seedlings in afforested areas have been reported as damaged or uprooted [18]. In urban areas, visible conflicts involving wild boars are becoming more common. This species has established itself in the outskirts of major European cities, leading to negative consequences associated with its presence in these environments. However, the most critical contemporary issue is related to the transmission of African swine fever (ASF). Although African swine fever has been present in parts of Europe since the 20th century, its large-scale spread across Eastern Europe began after 2007. The spread is continuing throughout Asia, including China and other southeastern regions [19,20]. Therefore, ASF has become a global concern, negatively impacting wild boar populations [21] and, even more significantly, imposing severe restrictions on the domestic pig farming industry, resulting in substantial economic losses [22].

Given the increasing rate of wildlife conflicts, wild boar can be classified as overabundant [23]. At the same time, the number of active hunters is decreasing in many countries, significantly weakening the primary mechanism for population control—hunting—and contributing to the unprecedented population growth [2]. Although hunting remains the main control method for managing wild boar populations, the species’ growing reproductive capacity and its adaptation to changing environments, including urban areas, complicate population control efforts [24,25]. In countries such as France, there is an urgent need for new, targeted hunting models that are better aligned with current social and ecological conditions, including principles of animal welfare [26]. This issue is particularly urgent in urban and suburban areas, where hunting opportunities face restrictions due to legal and safety regulations. Effective population management thus requires a combination of selective capture and hunting methods with strategic targeting of specific age and sex categories [27]. In this study, selectivity refers to the ability of a control method to target wild boar specifically while minimizing impacts on non-target species.

Considering the above, it is evident that reducing overabundant wild boar populations through direct hunting alone is insufficient [2]. The continued population increase demands the exploration of additional, less commonly applied control methods such as trap cages, capture systems, various types of targeted shooting, or poison baiting [28,29].

However, these alternative control measures raise several concerns compared to traditional hunting. These include not only questions of effectiveness, but also ethical implications and public acceptance. Therefore, this study aims to describe and evaluate global methods for reducing wild boar and feral pig populations. The specific objectives to assess are (i) the effectiveness of the applied methods; (ii) the selectivity of the applied measures; and (iii) compliance with animal welfare standards in the methods.

## 2. Materials and Methods

### 2.1. Literature Search Rules

**Figure 1:**
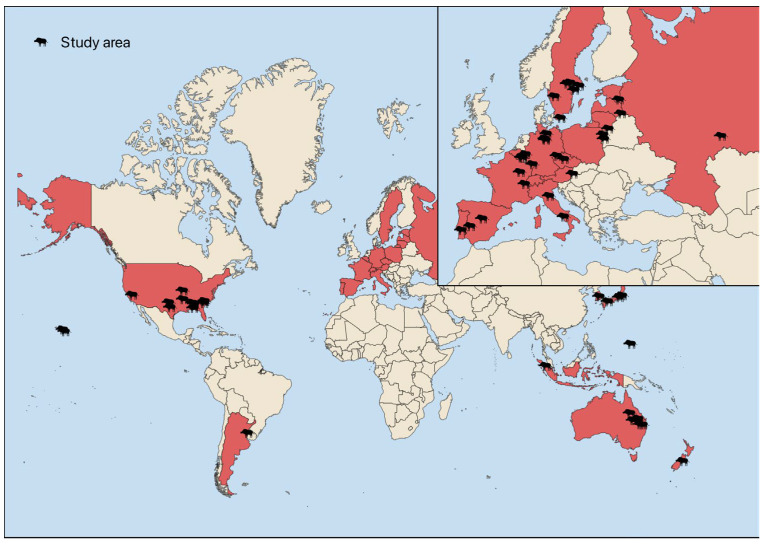
Map of analyzed studies. If exact coordinates were not provided, the approximate center of each described area is marked on the map.

This study was conducted in accordance with the PRISMA (Preferred Reporting Items for Systematic Reviews and Meta-Analyses) guidelines for systematic review reporting [30]. The PRISMA checklist is provided in Supplementary Material S1. The following was the central research question addressed: What is the current state of knowledge regarding the effectiveness, selectivity, and ethical compliance of the most used methods for reducing wild boar and feral pig populations across different regions of the world? The geographical distribution of the included study areas is shown in Figure 1, where study locations are indicated by wild boar icons and countries are highlighted in red.

**Figure 2:**
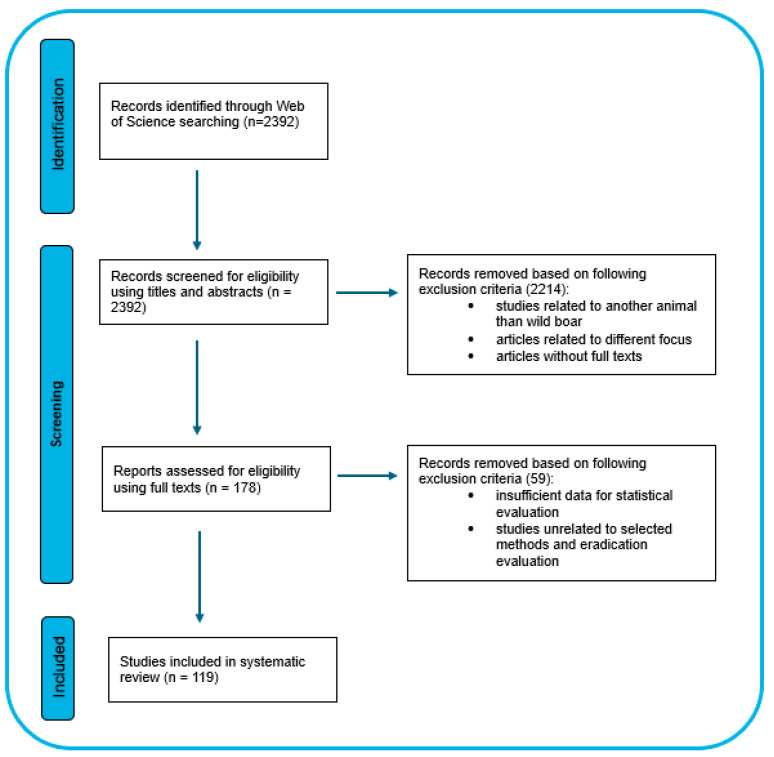
PRISMA (Preferred Reporting Items for Systematic Reviews and Meta-Analyses) flow diagram illustrating the process of identification, screening, and inclusion of studies in the systematic review (adapted from [30]).

A systematic search was conducted using the Web of Science citation database, which includes peer-reviewed journals in biology, ecology, and environmental sciences. The last search was performed on 11 November 2025. The review included studies focusing on *Sus scrofa* (wild or feral pig/boar) and evaluated methods of population eradication and their effectiveness. Search queries were constructed by combining search terms and key elements using Boolean operators (AND, OR). The formulation of the Boolean operator was (“wild boar*” OR “feral pig*” OR “feral hog*” OR “wild pig*”) AND (“population* reduction*” OR “population* eradication*” OR trap* OR “drop net” OR capture* OR hunt* OR cull*). Studies excluded after full-text screening were removed due to insufficient data or lack of relevance to the study objectives (*n* = 59) (see Figure 2). The selection process of the retrieved records was carried out in two screening phases, as illustrated in Figure 2. A detailed description of the methods is provided in Supplementary File (S2). The whole dataset of the 119 included studies is available as a separate Supplementary File (S3). These studies contain 181 independent experiments. The review included studies from regions and time periods both with and without the presence of African swine fever. The objective was to evaluate general wild boar population control methods rather than African swine fever-specific management strategies. Only peer-reviewed articles published in English were included to ensure consistency in data extraction and interpretation across studies.

This systematic review was conducted in accordance with the PRISMA (Preferred Reporting Items for Systematic Reviews and Meta-Analyses) guidelines. The review was not registered, and no protocol was prepared. Due to substantial heterogeneity in study design, interventions, and outcome reporting, no formal meta-analysis was performed. Consequently, analyses related to heterogeneity (e.g., subgroup analyses) and sensitivity analyses were not conducted. In addition, risk of bias across studies (including reporting bias) was not formally assessed, and the certainty of evidence was not evaluated, as the review was based on a qualitative synthesis of heterogeneous studies.

### 2.2. Statistical Analysis

Due to insufficient data in the available studies, a more detailed analysis of the effectiveness of individual methods (shooting, trapping, toxic baiting) was not possible. Many studies did not provide sufficiently specific information regarding the types of traps, toxicants, hunting practices or local conditions, or specific technical and environmental factors, which complicated a more detailed breakdown of these methods in the analysis.

The effectiveness assessment of selected methods was divided into an analysis of instantaneous effectiveness, i.e., the effectiveness in hunted/trapped individuals per hour, during eradication attempts, and overall effectiveness, which was computed from reported data as the total relative number of eradicated individuals (in %) divided by experiment duration in months. Both metrics were statistically compared between selected eradication methods by the Kruskal–Wallis test. In both cases, only methods that had at least three valid records were included in the analysis. The results are depicted by bar plots.

For trapping, the relationship between the total relative number of eradicated individuals and experiment duration (in months) was assessed by linear regression. The results of this analysis are depicted by a scatter plot with a trend line and a 95% confidence belt.

The comparison of total relative population reduction for selected eradication methods and selectivity variants (methods with at least three records for both selectivity variants) was conducted. For each variant, the values between selectivity and non-selectivity were compared using the Wilcoxon rank-sum test (as the assumption of normality for the *t*-test was not met in any case).

Lastly, the comparison of the total relative number of eradicated individuals between studies, which assessed or did not assess animal welfare/stress, was performed using the Wilcoxon rank-sum test (as the assumption of normality, tested by the Shapiro–Wilk test, for the t-test was not met). The results are depicted by a bar plot showing mean values with 95% CI.

All statistical analyses were performed in R software (version 4.5.2). Plots were created using the ggplot2 package [31]. The overview map (Figure 1) was created in QGIS software using the OpenDataSoft World Administrative Boundaries dataset (available on https://public.opendatasoft.com/explore/dataset/world-administrative-boundaries/export/ (accessed on 29 November 2025)). For all statistical procedures, an alpha level of 0.05 was selected.

## 3. Results

### 3.1. Overview of Wild Boar Population Reduction Methods

**Figure 3:**
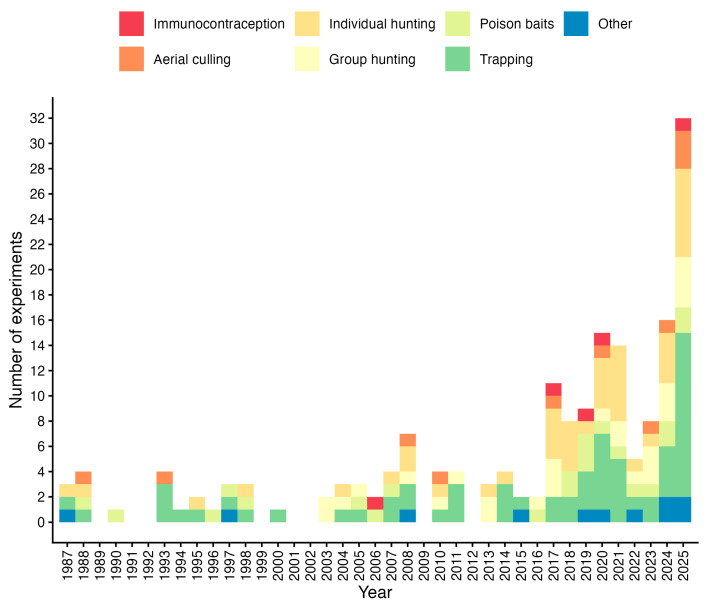
Basic overview of the numbers of experiments dealing with wild boar population reduction during the study period. “Other” includes mixed or combined approaches that could not be assigned to a single control method category.

The basic evaluation of the included literature sources shows that trapping was the most frequently studied method for wild boar population reduction (62 out of 181 analyzed experiments; see Figure 3). In contrast, the least attention was paid to aerial culling (11 experiments) and immunocontraception (5 experiments), which are regionally specific and only based on exceptions not acceptable for Europe. However, there has been a significant increase in the number of relevant experiments in recent years, particularly between 2017 and 2025. During this period, research primarily focused on wild boar trapping (38 experiments), while individual hunting was also frequently studied (32 experiments). Contrastingly, the fewest studies during this period addressed aerial culling (only nine experiments), reflecting the overall trend observed across the full study period.

### 3.2. Eradication Effectiveness of Methods

**Figure 4:**
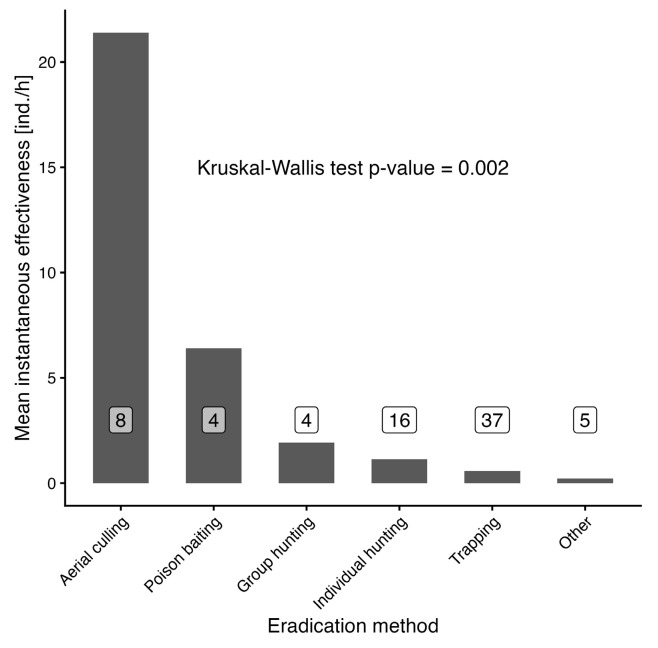
Comparison of mean instantaneous effectiveness for selected eradication methods. Numbers in white labels stand for number of observations for each category.

The analysis of instantaneous eradication effectiveness for selected methods showed the highest mean values for aerial culling (21.4 individuals per hour), with the following methods showing much lower numbers: poison baiting (6.4 ind./h), group hunting (1.9 ind./h), individual hunting (1.1 ind./h), trapping (0.6 ind./h), and other studied methods together (0.2 ind./h). The Kruskal–Wallis test indicated a significant result (Chi-squared = 19.12, df = 5, *p* = 0.002). The difference from aerial culling is apparent, but a definitive separation from other methods was not established through multiple comparisons (see Figure 4).

### 3.3. Eradication of Subpopulation in Percents

**Figure 5:**
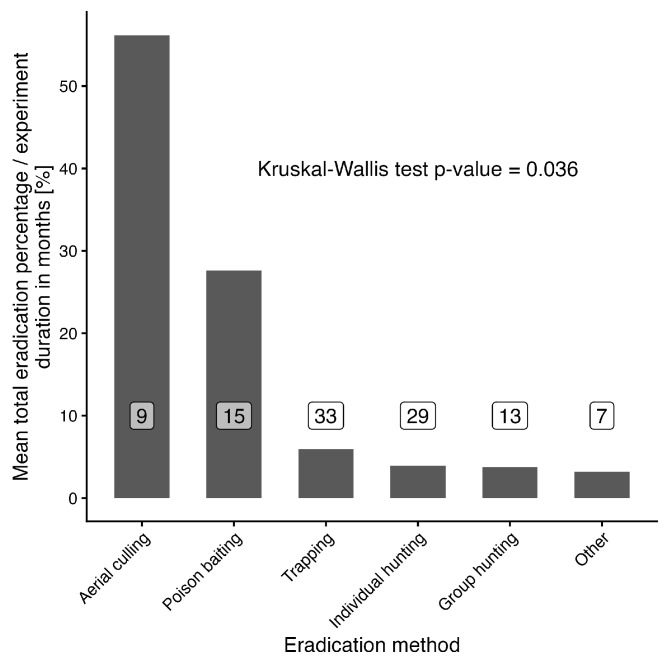
Comparison of mean overall effectiveness for selected eradication methods. Numbers in white labels stand for number of observations for each category.

A similar analysis was also performed for overall effectiveness, i.e., relative numbers of eradicated individuals (in %) divided by the eradication duration (in months; see Figure 5). The highest mean values were observed for aerial culling (56.2%/month) followed by poison baiting (27.6%/month) as in the previous case, followed by trapping (6.0%/month), individual hunting (4.0%/month), group hunting (3.7%/month), and other methods together (3.2%/month). Similarly, as before, the Kruskal–Wallis test showed a significant result (Chi-squared = 11.94, df = 5, *p* = 0.036), as the difference in aerial culling is also evident. Nevertheless, multiple comparisons did not show a clear separation from other methods, presumably due to relatively low observation counts (analyzed experiments) for some methods.

### 3.4. Population Reduction Based on Experiment Duration

**Figure 6:**
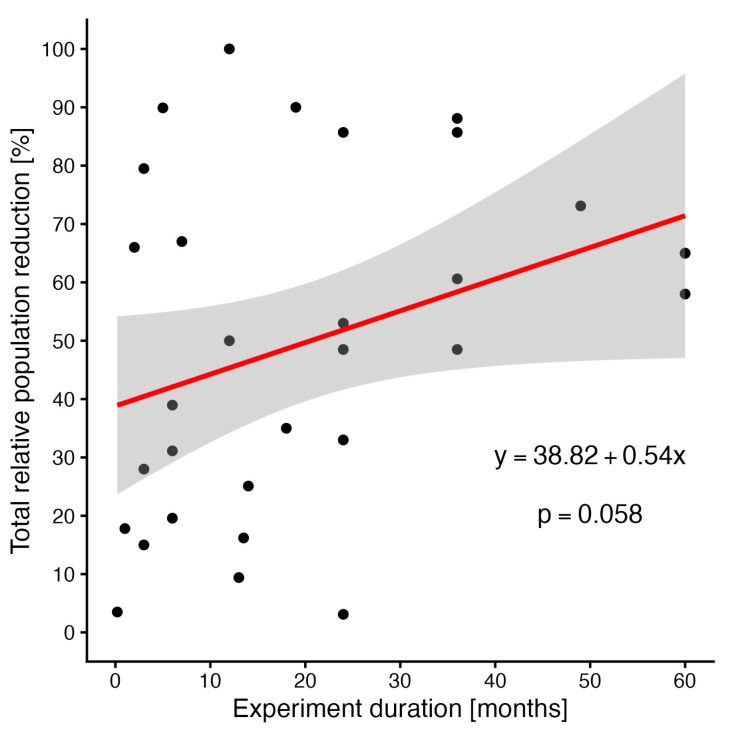
The relationship between total relative population reduction and experiment duration. The red line stands for the result of linear regression, and the shaded area for its 95% confidence belt.

For trapping, a separate analysis of relationships between the total relative population reduction and the duration of the experiment (eradication effort) was performed (see Figure 6). Linear regression showed an increasing trend line; however, it was marginally insignificant (*p* = 0.058), perhaps due to the relatively high variance in data. Two far outliers (data for 192 months and 96 months) were excluded from the analysis.

In one case, all individuals in the area were trapped during 12 months of the experiment, followed by 90% of individuals trapped in 19 months, or 89.9% of individuals that were trapped in only five months. On the other hand, one study showed that only 3.1% of individuals were trapped in 24 months. The longest experiment assessing trapping with available data lasted 192 months, with a ratio of trapped individuals of 27.5%; conversely, the shortest experiment, which used trapping, lasted 0.2 months and showed a ratio of trapped individuals of 3.5%.

### 3.5. Selectivity of Methods

**Figure 7:**
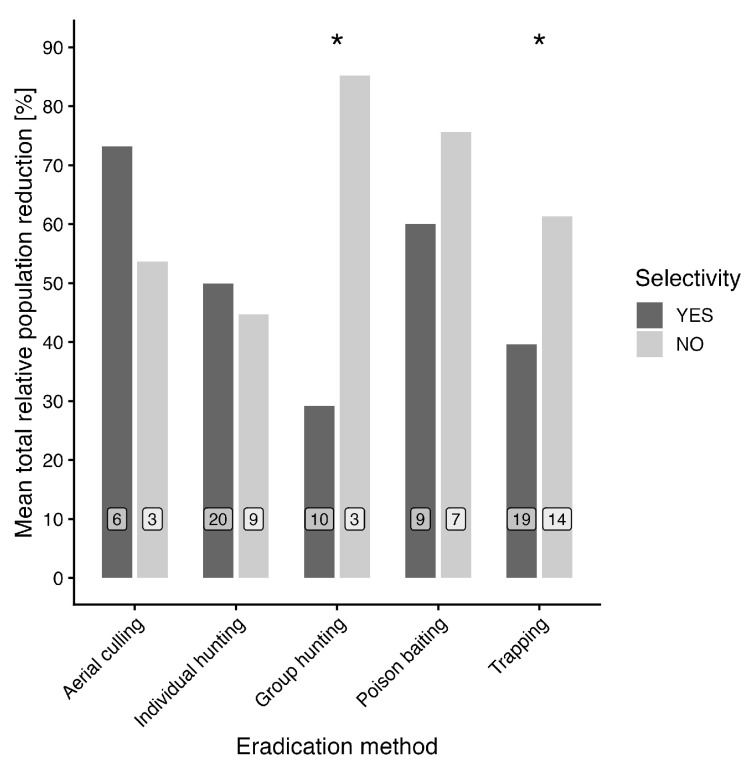
Total relative population reduction for selected eradication methods and hunting selectivity variants. Here, selectivity refers to the consideration of non-target species in the evaluated studies. The asterisks above respective variants symbolize significant difference for the comparison of total relative population reduction between experiments with hunting selectivity/non-selectivity. Numbers in white labels stand for the number of observations for each category.

In this analysis, studies were categorized based on whether they explicitly evaluated interspecific selectivity. In some cases, lower effectiveness in selective approaches may reflect a stronger emphasis on minimizing impacts on non-target species, particularly in methods such as trapping and baiting. The comparison of total relative population reduction for selected methods between selectivity variants showed significant results for trapping and group hunting (see Figure 7). In the case of trapping, experiments without selective trapping showed a mean value of 61.4%, compared to experiments with selective trapping, where the mean value of the total relative population reduction was 39.6%. In the case of group hunting, selective hunting showed a total relative population reduction of 29.2%, while in the case of non-selective hunting, a total relative population reduction of 85.2% was observed. Only variants with at least three records for both selectivity variants were involved in the analysis.

### 3.6. Aspect of Welfare

**Figure 8:**
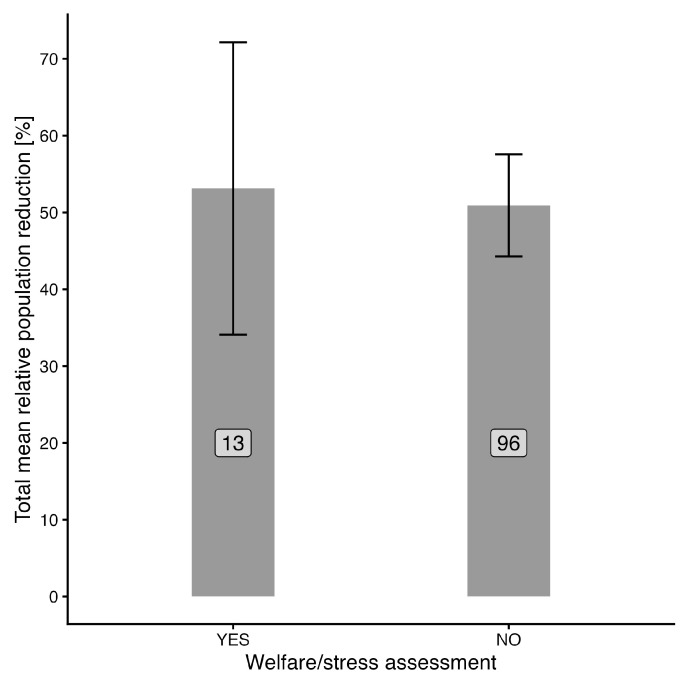
Comparison of total mean population reduction between experiments assessing and not assessing animal welfare or stress. Bars depict the mean value, and error bars mean ± 95% CI. Numbers in white labels stand for the number of observations for each category.

Finally, the comparison of total relative population reduction between experiments, which considered welfare/stress assessment of wild boars, was performed (see Figure 8). The Wilcoxon rank-sum test showed an insignificant result (W = 637.5, *p* = 0.9) for the comparison of total relative population reduction between experiments with and without welfare or stress assessment. The mean value for experiments on welfare/stress was 53.1% (95% CI ± 19%), while for other experiments it was 50.9% (95% CI ± 6.6%).

## 4. Discussion

This systematic review with quantitative synthesis of previously published studies shows that most research on wild boar population control originates from Europe. At the same time, efforts to address this pressing issue have become evident in recent years. Scientific research seems to be responding to the wild boar population explosion, as demonstrated by a marked acceleration in studies focusing on population control methods between 2017 and 2024. This reflects the high level of conflict with the species in the European context, including agricultural damage, traffic collisions, and ASF transmission [2,19,32].

Based on the results of the systematic review with quantitative synthesis, the effectiveness of various methods of wild boar eradication varies significantly. The most effective method appears to be aerial shooting, which is used predominantly in the USA and Australia, where both the geographical and legal contexts are open to this method [33]. The analysis confirmed that aerial shooting reached the highest mean values in both the instantaneous effectiveness (21.4 individuals/hour) and monthly eradication rate of the population (56.2%) and was significantly higher compared to other methods used. In Europe, however, the applicability of this approach is constrained by legal regulations, fragmented land ownership, high levels of urbanization, and public opinion, including the welfare of hunted individuals [1].

Incorporated analyses also showed moderate effectiveness of poison baiting (27.6%/month) and trapping (6.0%/month), both of which showed considerably lower values than aerial culling. However, the application of poison baiting is generally not permitted under current European legislation for the same reasons as aerial shooting [1]. Driven hunting (3.7%/month) and individual hunting (4.0%/month) ranked even lower in overall efficiency, suggesting that traditional methods alone are not sufficient for population reduction at the necessary scale. The effectiveness of other methods, such as driven hunts, individual shooting, or trapping, often varies and depends on local conditions, seasonality, and the targeted demographic group. This is particularly evident in Europe, where the wild boar population density continues to rise [2] despite ongoing hunting efforts. These efforts, nevertheless, primarily employ traditional hunting methods, including individual hunting and driven hunts. In addition to biological effectiveness, cost-effectiveness is an important consideration, as different methods vary substantially in economic and logistical demands [17].

The selectivity of the method can enhance the effectiveness of reducing wild boar and feral pig populations. As demonstrated by the systematic review with quantitative synthesis results, aerial shooting appears to be the most selective and simultaneously appears to be the most effective method. Aerial shooting is primarily targeted only at feral pigs [34] or at other ungulate pest species [35]. Individual hunting can also be selective; however, hunters often shoot other ungulate species according to current opportunities. On the other hand, selectivity is not guaranteed in the case of trapping and poison baiting. In both cases, the selectivity of these methods could be affected by the type of bait. This can be complicated for wild boars due to their opportunistic feeding behavior [36]. Therefore, the poison baiting is not selective in the most cases [37,38,39], which is the greatest disadvantage for the eradication method, exacerbating potential risks to other wildlife species, including protected ones. In this context, trapping is also challenging. Selectivity can hardly be ensured by the type of trap or bait alone, because in both cases, the device may be triggered by a non-target species due to the more elaborate construction required for trapping wild boar. A potential solution lies in the use of a remotely operated triggering mechanism based on live camera footage. In such systems, the operator activates the trap only when wild boars are positively identified—a method that has already been tested by Verde et al. [40].

Selectivity should also be considered in an intraspecies context when it comes to population reduction. For instance, Escobar-González et al. [27] reported that selective female hunting has a significantly greater impact on the population’s reproductive capacity than non-selective shooting. When managing wild boar populations, it is crucial to focus on selectivity to improve effectiveness. This can be best achieved by targeting adult females, as they tend to produce more offspring than younger boars, a factor closely related to their body weight. Under European conditions, sows weighing approximately 60 kg or more can conceive 8 to 12 piglets, whereas females weighing up to 40 kg typically produce only 2 to 4 piglets [11]. This rapidly accelerates the potential for further population growth. On the other hand, it is essential to strictly adhere to ethical principles, including avoiding the hunting of heavily pregnant females or those with newborn piglets that are still fully dependent on them.

The present results show that studies including assessments of stress or welfare did not differ in population reduction effectiveness from studies without such assessments. The average values were nearly identical (53.1% vs. 50.9%; *p* = 0.9), indicating no detectable relationship in the available dataset. Therefore, it is not possible to conclude that welfare-focused approaches enhance or reduce effectiveness. Only a small number of studies (13 of 181 analyzed) explicitly considered welfare, which highlights a substantial research gap [1]. Although traps, for example, may be effective and considered relatively humane, concerns remain about high stress levels in animals that may be held for prolonged periods [33]. Aerial shooting, while highly effective, is ethically controversial and generally unacceptable and requires special consideration only in situations where other methods have failed. Additionally, there is no standardized methodology for assessing the stress and welfare impacts on boars, which makes relevant comparisons impossible. This is notably critical due to increasing demands for humane treatment of animals and public pressure for more ethical approaches to wildlife management [26], including non-lethal measures, especially in urbanized areas [41].

Interpretation of the results is constrained by substantial variability in study design, limited sample sizes for some methods, and the absence of standardized criteria for evaluating effectiveness and welfare impacts. These factors reduce comparability across studies and highlight the need for methodological harmonization. This review has several limitations related to the review process, including the use of a single database (Web of Science) and restriction to English-language publications, which may have introduced selection bias and limited the comprehensiveness of the evidence base.

Furthermore, temporal changes in effectiveness were not clearly identified in the available data, likely due to local variability and increasing population densities. In other studies, changes in the effectiveness of methods over time are influenced not only by technical improvements but also by the growing densities of wild boar populations, which may “mask” the success of interventions [2]. This complicates year-to-year and inter-regional comparisons, underlining the need to standardize both methodological and demographic evaluation criteria [6].

Regression analysis of trapping success in relation to eradication duration indicated a positive but statistically insignificant trend (*p* = 0.058), likely influenced by high variability among studies. Certain cases demonstrated near-complete eradication within short timeframes (e.g., 90% reduction in 5 months), whereas others reported minimal results even over extended periods. This variance underscores the importance of local ecological and logistical contexts in determining success. It is also essential to emphasize that, for wild boar population reduction to be effective, it must be implemented over an extended period and across a broader spatial scale. This is often a limitation of these analyzed studies, which typically focus on reducing wild boar numbers at a local scale [2,42,43,44]. The species’ high reproductive potential and ecological plasticity contribute to its capacity for rapid population recovery, even following intensive control efforts [12].

However, hunting efficiency is influenced not only by applied methods but also by a range of other factors. Setting aside the social structure and local wild boar population density, the surrounding environment becomes a key factor, especially from the perspective of availability of shelter and, above all, food resources. Seasonality plays a significant role as well; for example, trapping tends to be more successful in winter, when food availability is scarce, and animals are more likely to enter the traps. Additionally, individual hunting combined with supplementary feeding can be effective [25,45]. Nevertheless, in Central European conditions, hunting success during the winter is affected by the mast years of oak or beech [11], when the wild boar prefer natural food sources over supplementary feeding. Moreover, hunting philosophy may pose a significant challenge in reducing wild boar populations, even though relatively effective methods such as trapping are available. Recreational hunters may not always fully engage in wild boar population reduction, which has been identified as a potential limitation in management effectiveness [46]. Lastly, public perception of certain control methods—particularly invasive ones—must be considered. Successful wild boar population control in human-dominated landscapes will require not only technical effectiveness but also ethical, legal, and social acceptance of the proposed solutions [46,47,48]. Therefore, effective population control of wild boar and other common ungulate species depends on identifying suitable methods. Trapping appears to offer a reasonable compromise between effectiveness, selectivity, and animal welfare. The feasibility of the proposed management recommendations varies among regions depending on legal frameworks, public acceptance, and landscape conditions. Therefore, their implementation should be adapted to local policy and management contexts. Further research should primarily focus on public engagement to ensure broader acceptance of the methods and explore ways to motivate hunters, or to increase the involvement of professional wildlife managers in achieving population reduction.

## 5. Conclusions

The collected systematic review with quantitative synthesis confirmed an increase in research activities addressing effective reduction in wild boar and feral pigs, mirroring the response to the recent population increase. However, the analysis did not identify an ideal solution, highlighting the need for a combination of methods adapted to specific ecological and social contexts. The systematic review with quantitative synthesis confirmed that the most effective practices, such as aerial shooting and poison baiting, remain geographically limited. In the European context, only traditional methods of population control are legal and acceptable, with moderate and highly context-dependent effectiveness, which points to a significant research gap in wild boar population control methods despite the recent increase in research interest. Studies incorporating animal welfare or stress assessments did not differ in their effectiveness from studies without such assessments, as the observed values were very similar, and the statistical test showed no significant difference (*p* > 0.05). Consequently, the current evidence does not support any clear relationship between welfare considerations and eradication success. Nevertheless, welfare remains an important aspect of population management, particularly given increasing public expectations for humane wildlife control. To achieve effective, selective, and ethically acceptable wild boar population control, it is necessary to:

1. Standardize methodologies for evaluating both effectiveness and welfare.
2. Combine multiple methods (e.g., trapping and selective shooting) based on local conditions and resources.
3. Target adult females during the non-reproductive season, which may improve the long-term effectiveness of population control based on available studies.
4. Promote public acceptance of ungulate population reduction and develop appropriate incentive mechanisms for hunters.

Interpretation of the results is limited by heterogeneity in study design, variable data quality, and insufficient standardization of welfare and effectiveness metrics across studies.

## Data Availability

The data supporting the findings of this study are available in Supplementary File S3, which contains the full dataset of analyzed studies.

## Supplementary Materials

The following supporting information can be downloaded at https://www.mdpi.com/article/10.3390/ani16071023/s1: S1: PRISMA 2020 Checklist; S2: Detailed description of the methodology; S3: Dataset of analyzed studies.
