# Supplementary material for: Balancing Effectiveness and Ethics: Global Systematic Review of Sus scrofa Population Control Methods
Source: Animals (Basel). 2026 Mar 27;16(7):1023. doi: 10.3390/ani16071023 (PMC13072410; doi:10.3390/ani16071023)
Supplement: Supplementary file 1 [file animals-16-01023-s001.zip › Supplementary file_S2_Detailed_description_of_the_methodology.pdf]

All available types of studies were eligible for inclusion if they met the following criteria: (i) published in English; (ii) focused on the species *Sus scrofa* (wild or feral pig/boar); (iii) included individuals of various age and sex categories; and (iv) evaluated methods of population eradication and their effectiveness. Studies not directly addressing population reduction or lacking sufficient detail regarding eradication methods were excluded from the synthesis.

The following Boolean expression was used to identify relevant studies: *("wild boar" OR "feral pig*" OR "feral hog*" OR "wild pig*") AND ("population* reduction*" OR "population* eradication*" OR trap* OR "drop net" OR captur* OR hunt* OR cull*). The query was applied to the “Title,” “Abstract,” and “Keyword Plus” fields. No date restrictions were imposed. Only articles published in English were included. Grey literature was excluded due to concerns regarding verifiability and the absence of peer review.

Study selection was performed in two stages. First, two independent reviewers screened the titles and abstracts of all retrieved records. Disagreements were resolved through discussion or, if necessary, by consulting a third reviewer. Full-text articles of potentially relevant studies were then assessed for eligibility. The screening process and data management were carried out using Microsoft Excel.

Data extraction was conducted independently by two reviewers using a standardized, pre-designed data extraction form implemented in Microsoft Excel. The form was developed by a third reviewer and validated through consensus within the review team. Any discrepancies between the two reviewers were resolved through discussion.

The following information were extracted from each included study: article title, authors, type of study, year, sample size, area size (in hectares), eradication method, selectivity of the method (yes/no), demographic characteristics of removed individuals (age), assessment of welfare and stress indicators (yes/no), effectiveness of removal (individuals per hour), percentage population reduction, study duration (in months), and simplified categorization. Where data were not available, the field was marked as “NA.” Where feasible, effectiveness and reduction were calculated from raw data.

The risk of bias was assessed at the study level by two independent reviewers. Key aspects of methodological quality were evaluated, including study design, control of confounding variables, and completeness of outcome reporting. In cases of disagreement, a consensus was reached through consultation with a third reviewer. The specific assessment tool to be applied will be determined in consultation with a statistician.
